# Novel Intronic RNA Structures Contribute to Maintenance of Phenotype in *Saccharomyces cerevisiae*

**DOI:** 10.1534/genetics.115.185363

**Published:** 2016-05-18

**Authors:** Katarzyna B. Hooks, Samina Naseeb, Steven Parker, Sam Griffiths-Jones, Daniela Delneri

**Affiliations:** Faculty of Life Sciences, University of Manchester, M13 9PT, United Kingdom

**Keywords:** introns, ncRNA, RNA structure, yeast

## Abstract

The *Saccharomyces cerevisiae* genome has undergone extensive intron loss during its evolutionary history. It has been suggested that the few remaining introns (in only 5% of protein-coding genes) are retained because of their impact on function under stress conditions. Here, we explore the possibility that novel noncoding RNA structures (ncRNAs) are embedded within intronic sequences and are contributing to phenotype and intron retention in yeast. We employed *de novo* RNA structure prediction tools to screen intronic sequences in *S. cerevisiae* and 36 other fungi. We identified and validated 19 new intronic RNAs via RNA sequencing (RNA-seq) and RT-PCR. Contrary to the common belief that excised introns are rapidly degraded, we found that, in six cases, the excised introns were maintained intact in the cells. In another two cases we showed that the ncRNAs were further processed from their introns. RNA-seq analysis confirmed that introns in ribosomal protein genes are more highly expressed when they contain predicted RNA structures. We deleted the novel intronic RNA structure within the *GLC7* intron and showed that this region, rather than the intron itself, is responsible for the cell’s ability to respond to salt stress. We also showed a direct association between the in *cis* presence of the intronic RNA and *GLC7* expression. Overall, these data support the notion that some introns may have been maintained in the genome because they harbor functional RNA structures.

THERE are three main theories regarding the origin of introns: “Introns Late,” “Introns Early,” and “Introns First” (Jeffares *et al.* 2006). The Introns Late and Introns Early theories suggest that introns arose within the eukaryotic lineage, and before the Prokaryota-Eukaryota split, respectively, whereas Introns First implies that these noncoding sequences appeared before protein-coding genes. Introns are maintained, lost or gained with different rates within different eukaryotic lineages. The recent evolution of the yeast genome has seen widespread intron loss, with the result that only 5% of *Saccharomyces cerevisiae* genes contain introns (Dujon 2006). It has been proposed that small organisms with a large effective population size, such as *S. cerevisiae*, have seen deleterious introns gradually eliminated from the genome (Lynch 2002). This raises the question of why some introns are maintained in the yeast genome and, furthermore, whether they are functionally relevant.

Recent studies indicate that *S. cerevisiae* introns increase fitness under stress (Parenteau *et al.* 2008), aid ribosome assembly, and regulate expression of the paralogous copy of the gene (Parenteau *et al.* 2011). Introns may contain regulatory sequences and structures that affect splicing or expression of their host genes. In the *HAC1* transcript, short RNA hairpins define the intron boundaries (Sidrauski and Walter 1997). Splicing of the *YRA1* messenger RNA (mRNA) regulates its export from the nucleus (Preker *et al.* 2002; Rodriguez-Navarro *et al.* 2002). Described RNA structures in pre-mRNA transcripts of *RPL30* (Li *et al.* 1996) and *RPS14B* (Fewell and Woolford 1999) regulate splicing of their host genes. Intronic hairpins in *RPL18A* and *RPS22B* pre-mRNAs are recognized by RNase III Rnt1p and promote mRNA degradation (Danin-Kreiselman *et al.* 2003). The *RPS17B* intron contains a RNA-based splicing enhancer that physically decreases the distance between the splice sites (Rogic *et al.* 2008). RNA structures in the introns of *RPS9A* and *RPS9B* transcripts are vital components of an autoregulatory circuit (Plocik and Guthrie 2012).

The cases described above are examples where introns function in *cis*; intronic sequences can also act in *trans*. In vertebrates, introns frequently harbor functional noncoding RNAs (ncRNAs). Tiling arrays and deep RNA sequencing (RNA-seq) have revealed the existence of many novel intronic transcripts, most of which have no known function (Cheng *et al.* 2005; Mercer *et al.* 2008). In addition, a recent study searching for RNAs bound by the chromatin-modifying polycomb complex raised the possibility that intronic ncRNAs can be used to guide chromatin modifications that influence gene expression in a manner analogous to some long intergenic ncRNAs (Guil *et al.* 2012). In vertebrates, there are also well-characterized examples of intronic ncRNAs such as transfer RNAs (tRNAs), small nucleolar RNAs (snoRNAs), and microRNAs (miRNAs) (Kim and Kim 2007). However, in *S. cerevisiae*, well-known intronic ncRNAs classes are not prevalent: miRNAs are nonexistent, due to the loss of pre-miRNA processing enzymes (Drinnenberg *et al.* 2009); intronic snoRNAs have been mostly “deintronized” (Mitrovich *et al.* 2010); and all tRNAs are present outside introns. Even though many ncRNAs have been found in *S. cerevisiae*, such as stable unannotated transcripts (SUTs) and cryptic unstable transcripts (CUTs) (Wu *et al.* 2012), only 2% of the instances of these classes overlap with introns (Xu *et al.* 2009). It is commonly believed that splicing in higher eukaryotes increases protein diversity by providing multiple mRNAs from a single locus. However, alternative splicing in *S. cerevisiae* has been shown only for transcripts from the genes *SRC1* (Davis *et al.* 2000; Grund *et al.* 2008), *PTC7* (Juneau *et al.* 2009), and *MTR2* (Davis *et al.* 2000; Preker *et al.* 2002). Since alternative splicing in *S. cerevisiae* is rare and there are few typical intronic ncRNAs, we hypothesize that functional yeast introns may have been retained because they contain novel ncRNAs or pre-mRNA structures.

In order to discover potential functional RNA structures within introns in *S. cerevisiae*, we performed a computational screen for novel structures using intron orthologs from 36 fungal species and employing three *de novo* RNA structure prediction tools. The screen identified 19 introns containing potential RNA structures, and we validated the expression and processing of a subset by RT-PCR. We showed that six introns tested are maintained in the cell after splicing and two contain candidate novel ncRNAs. A novel RNA structure within the *GLC7* intron, rather than the whole intron (Juneau *et al.* 2006; Parenteau *et al.* 2008), is responsible for the cell’s ability to respond to salt stress, by altering the gene expression.

## Materials and Methods

### Intron alignments

Sequences of intron-containing genes were extracted from the *Saccharomyces* Genome Database (SGD, http://www.yeastgenome.org/). Genes orthologous to intron-containing genes from *S. cerevisiae* were identified in 36 fungal genome sequences (Supplemental Material, Table S1) using TBLASTX, with the coding gene sequence as the query and with the following settings: -E e-6 -qframe 1 -hspsepsmax 1000 -topcomboN 1. We collected the sequences of putative orthologs with at least 65% query coverage, to which 1000 bp and 300 bp of flanking sequence were added to the 5′ and 3′ ends of each hit. The putative ortholog gene sequences were then searched for the presence of the orthologous intron using BLASTN with options: -E 0.1 -W 3 -hspsepsmax 1000. The best hit (with the lowest *e*-value and confirmed by manual inspection) was retained for each intron in each species.

### RNA structure predictions

Three structure prediction programs were used: CMfinder (v 0.2), RNAz (v 2.0), and Evofold (v 7b). Sequences of orthologous introns were used for predictions with CMfinder as described in Torarinsson *et al.* (2008). CMfinder was run with settings: -n 5 -m 30 -M 100 and -s 2 -n 5 -m 40 -M 100, and identified motifs were extended using the CombMotif.pl procedure. Motifs with a composite score of *r* > 5 and folding free energy of < −5 kcal⋅mol^−1^ were considered as putative positives. For RNAz and EvoFold predictions, intron sequences were first aligned with mLAGAN. Structure prediction by RNAz was performed according to the manual (http://www.tbi.univie.ac.at/∼wash/RNAz/manual.pdf). The, rnazWindow.pl script was used to slice the alignments with the following options: –max-gap=0.25 –min-id=30 –max-seqs=6. RNAz was then run on the forward strand of the gapped alignments (options: –forward –g –p 0) and sequences with probability *P* > 0.5 were considered as putative positives. For EvoFold predictions, the required phylogenetic tree containing the species present in the intron alignments was derived by pruning the tree presented by Medina *et al.* (2011), and the subsequent structure predictions were predicted using default parameters. We employed a threshold value of 10 for the log-odds ratio of the likelihood of the region under the structure model and background model. The complete list of all predictions can be found in File S1. To extend the phylogenetic range of the RNA predictions, BLAST and INFERNAL 1.0.2 (Nawrocki *et al.* 2009) were used to re-search all fungal genomes in an iterative process, based on the Rfam approach (Gardner *et al.* 2010) and as described previously (Hooks and Griffiths-Jones 2011).

### Strains and media

Intron sequence replacement strains were engineered from the BY4743 (*MAT***a**/α, *his3*Δ1/*his3*Δ*1*, *leu2*Δ*0*/*leu2*Δ*0*, *lys2*Δ*0*/*LYS2*, *MET15*/*met15*Δ*0*, and *ura3*Δ*0*/*ura3*Δ*0*), BY4742 (*MAT*α, *his3*Δ*1*, *leu2*Δ*0*, *lys2*Δ*0*, and *ura3*Δ*0*) and BY4741 (*MAT***a**, *his3*Δ*1*, *leu2*Δ*0*, *met15*Δ*0*, and *ura3*Δ*0*) parental strains and maintained in YPD medium containing 2% (w/v) yeast extract, 1% (w/v) peptone, and 2% (w/v) glucose. The transformants were plated on solid YPD medium with 300 mg/ml geneticin (Gibco BRL) for kanMX selection and on 10 mg/ml phleomycin (Invitrogen) for pCre-ble selection. Mineral salts medium (F1 medium) used was of the following composition (grams/liter); (NH_4_)_2_SO_4_ (3.13), KH_2_PO_4_ (2), MgSO_4_⋅7H_2_O (0.55), NaCl (0.1), CaCl_2_⋅2H_2_O (0.09), and 2 ml of trace element solution per liter was added to it. The trace element solution used was of the following composition (grams/liter): ZnSO_4_⋅7H_2_O (0.7), CuSO_4_⋅5H_2_O (0.1), H_3_BO_3_ (0.1), and KI (0.1). The F1 was supplemented with 2% (w/v) glucose and 1.65 ml vitamin stock before use. The synthetic minimal SD medium (0.67% Bacto yeast nitrogen base without amino acids, 2% glucose) and F1 medium were also supplemented with required amino acids appropriate for the parental strain (Baganz *et al.* 1998). The media were filter sterilized.

### RNA extraction

The *S. cerevisiae* strain BY4741 was grown in 500 ml rich media (YPD) in 30° with shaking at 200 rpm to an absorbance of 0.5 at 600 nm. The RNA was extracted using Trizol (Invitrogen), precipitated in lithium chloride (Ambion), washed twice with 70% ethanol, and the pellet resuspended in dH_2_O. RNA concentration and quality was evaluated by measuring absorbance at 260 nm on a NanoDrop spectrometer ND-1000 (Thermo Scientific). The low molecular weight enriched RNA sample was obtained from total RNA as described in Catalanotto *et al.* (2002). Total RNA was used for RNA-seq and both total and low molecular weight enriched RNA was used for RT-PCR.

### RT-PCR

Complementary DNA (cDNA) was synthesized from 2 μg RNA of either total or low-molecular weight RNA using QuantiTect Reverse Transcription Kit (Qiagen) according to the manufacturer’s protocol. Fragments of cDNA corresponding to the predicted intronic RNA structure (ncRNA), whole intron of interest (intron), and exons surrounding the intron (mRNA) were amplified by PCR with BIOTAQ DNA Polymerase (Bioline) according to the supplier’s guidelines. The list of all primer sequences used can be found in File S2. The reaction mix was composed of 4 pmol of each primer and 20 ng of total or low molecular weight cDNA for each 10 μl of total reaction mixture. The cycling conditions were an initial denaturation for 5 min at 95°, 35 cycles of denaturation (45 sec, 94°), annealing (45 sec, 56°), and elongation (90 sec, 72°), followed by a final elongation for 5 min at 72°. Amplification of both cDNA using snR44 primers was used as a positive control. Genomic DNA extracted from BY4741 with Wizard Genomic DNA Purification Kit (Promega), according to the manufacturer’s protocol, was used as a positive control and water was used as a negative control. The PCR products were visualized by ethidium bromide staining on 1.2–3.5% agarose gels. For each predicted RNA structure, at least two independent PCR reactions with genomic DNA, total cDNA, low molecular weight cDNA, and snR44 positive control primers were performed in order to confirm expression.

### Northern hybridization

A total of 10 μg of the total RNA and 10 pmol of the oligonucleotides mimicking the intronic region of *GLC7* were loaded in RNA loading dye (Fermentas) onto separate lanes of a denaturating gel containing 36.5 mM MOPS, 9.1 mM sodium acetate, 0.9 mM EDTA, 2 M formaldehyde, 0.5 µg/ml ethidium bromide, and 1% agarose. RNA transfer, UV cross-linking, and Northern blotting were performed as previously described (Naseeb and Delneri 2012). [^32^P]-ATP end-labeled mixtures of two sense (to detect transcription from antisense strand) and two antisense oligonucleotides (to detect transcription from sense strand; all listed in File S2) were used as probes.

### Real-time PCR

The expression levels of the *GLC7* gene in the intron replacement mutant and BY4742 wild type grown in F1 media and in F1 + 0.9M NaCl were assessed by quantitative real-time PCR using the QuantiTect real time PCR kit (Qiagen, no. 204143). cDNA was extracted using QuantiTect Reverse Transcription Kit (Qiagen, no. 205311) according to the manufacturer’s manual. Real-time primers are listed in File S2. The PCR reactions were performed in triplicate for two independent biological replicas, as described previously (Naseeb and Delneri 2012). Relative normalized fold expression was calculated according to the ΔΔCt method using *ACT1* as a reference gene.

### RNA-seq

We used our previously generated RNA-seq data, deposited in Gene Expression Omnibus (GEO) under accession no. GSE58884 (Hooks *et al.* 2014). A total of 77,286,181 50-bp reads were filtered using the approach of Sasson and Michael (2010). Filtering left 45,520,779 reads with an average quality of >20, which were mapped to the *S. cerevisiae* genome (SacCer3) using Bowtie with settings –m 1 –v 2 (Langmead *et al.* 2009; Trapnell *et al.* 2009). A total of 25,254,315 reads with a maximum of two mismatches were mapped to the *S. cerevisiae* genome. To calculate average number of reads per intron [or the coding sequence (CDS)] [in reads per kilobase per million mapped reads (RPKM)], reads for each intron or CDS were summarized using featureCounts (Liao *et al.* 2014) and divided by the length of the feature in kilobases and the million reads mapped. The number of reads mapping to introns and their host genes is presented in File S3.

### Analysis of exosome target data

The cross-linking and analysis of cDNA (Complex Reads Analysis and Classification, CRAC) data presented by Schneider *et al.* (2012) were filtered for genes that contain introns. From the RNAseq data presented here, we calculated the number of reads in RPKM corresponding only to the ORFs for the same set of genes. For each individual CRAC experiment, the number of reads for each gene was normalized by the number of ORF reads for this gene from our RNA-seq data. The percentile rank of normalized values was calculated for each gene in each CRAC experiment, and then averaged across the 16 CRAC experiments (File S3). We considered genes with an average percentile rank with the top 10% to be preferentially bound by the exosome protein components.

### Deletion of predicted intronic RNA

In order to generate deletions of intron fragments or insertions into introns, the Cre-loxP system was used with a kanMX cassette flanked by loxP sites (Guldener *et al.* 1996). Deletion cassettes were amplified as described previously (Delneri *et al.* 2003). The *S. cerevisiae* BY4743 strain was transformed with 1 μg of each PCR product according to Gietz and Schiestl (2007). Selection of mutants and PCR confirmation were performed as described by Carter and Delneri (2010). The strains with the loxP-kanMX-loxP cassette were then transformed with the plasmid containing Cre-recombinase to excise the sequence between two loxP sites. Cre-recombinase was induced by culturing the cells overnight in the YP-raffinose medium and then for 2–3 hr in YP-galactose medium. KanMX excision was confirmed by PCR. Dissection of tetrads was performed using a Singer MSM 300 microdissector (Delneri *et al.* 2003). Haploids displaying the BY4742 metabolic background were chosen after series of cultures on SD solid medium lacking Lys, Met, or Ura.

The effect of the loxP deletion was to replace the 204-bp (*GLC7* ncRNA deletion mutant) and 140-bp (*GLC7* control deletion mutant) intronic regions with 139 bp of the loxP scar with the fragments of the transformation vector. We also constructed an insertion mutant with the 139-bp remnant of the cassette inserted into the middle of the predicted RNA structure without deleting any intron bases. All primers used in the creation of mutant strains are listed in File S2.

To perform the rescue experiment of the *GLC7* ncRNA deletion mutant, the wild-type (WT) *GLC7* ncRNA sequences were amplified and inserted in both sense and antisense orientations into a modified version of the pRS315 yeast shuttle vector (American Type Culture Collection, 77144), containing the constitutively expressed *TDH3* promoter and LEU2 auxotrophic marker. The pRS315 *TDH3* ncRNA recovery plasmids were transformed into BY4724 and the ncRNA deletion strain, following the method described in Gietz and Schiestl (2007). As a control, empty pRS315 was transformed into BY4742. Cells containing the plasmids were selected for by growth on SD −Leu media and successful PCR amplification of the plasmid.

### Growth rate assay

Growth properties of the BY4742 strain, the intron mutants, and rescue mutants were assessed by time course growth profiles obtained using a FLUOstar optima microplate reader. Cells were cultured to stationary phase in YPD or F1 medium. The OD was measured at 595 nm and the cultures were diluted to an OD at 595 nm of 0.1 with prewarmed YPD media, F1 media, or F1 media containing 0.9 M NaCl. Each of the 96-well plates was filled with 240 μl of diluted culture or media control. Absorbance measurements were taken every 5 min immediately after 1 min shaking. Growth curves were plotted and analyzed using R according to a modified version of the method specified previously (Norris *et al.* 2013). In brief, the area under each growth curve (AUC) was calculated by the pracma package from normalized absorbance data. Additionally, we fitted growth curves from data points taken every 30 min using grofit R package with default settings (Kahm *et al.* 2010). Maximum growth rate, lag phase, and maximum growth were calculated from the fitted curves.

### Competitive growth test

Competitive growth assays were performed in 8 ml media by adding an equal number of cells (2 × 10^5^ cells/ml) of a mutant strain and the BY4742 reference strain, which had the *HO* gene replaced with kanMX as a marker to facilitate selection between strains. The *GLC7*/reference competition was performed in F1 media containing 0.9 M NaCl. The cultures were grown at 30° and maintained in log phase by diluting each culture to 2 × 10^5^ cells/ml in fresh media every 12–24 hr until the generation number 37 ± 1 or 50 ± 2 was attained for F1 + 0.9 M NaCl or YPD media, respectively. The number of generations was calculated as described by Parenteau *et al.* (2008). When the appropriate generation number had been reached, ∼200 cells were plated on YPD media and after 2 days replicated onto YPD + geneticin. Cells were counted to obtain the ratio of mutant *vs.* reference strains.

### Data availability

Strains are available upon request. Figure S1, Figure S2, Figure S3, Figure S4, Figure S5, and Figure S6 contain images of full gels used for creating Figure 2. Figure S7 contains additional analysis of growth of *GLC7* mutants in F1 and F1 + NaCl media. Figure S8 contains negative result of the ncRNA rescue experiment. Table S1 contains a list of fungal genomes used in the study. File S1 contains a list of RNAz, Cmfinder, and EvoFold predictions for each intron. File S2 contains primer sequences and probes used in the study. File S3 contains expression of introns and coding sequences of host genes (in RPKM) and the average percentile from exosome targets data (CRAC) for each gene. This article reanalyzed two publically available data sets by Schneider *et al.* (2012), GEO accession no. GSE40046, and by Hooks *et al.* (2014), GEO accession no. GSE58884.

## Results

### Predictions of RNA structure within introns

**Figure 1:**
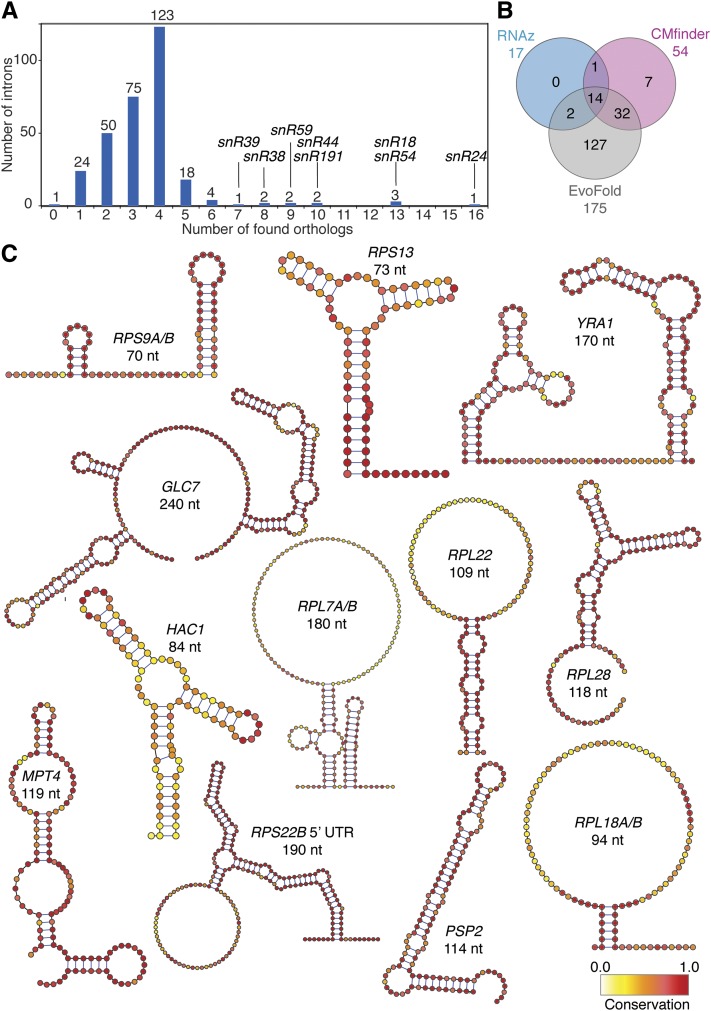
Predicted RNA structures. (A) For each intron of *S. cerevisiae*, the number of orthologous introns was counted among the 36 species included in the study. Plotted is the histogram of the number of introns (*y*-axis) yielding a specific number of orthologs (*x*-axis). Introns containing known snoRNAs are highlighted by name (snR). (B) Venn diagram showing the number of common RNA structures predicted by RNAz, Cmfinder, and EvoFold. For example, there are 14 introns with structures predicted by all programs. (C) Predicted consensus RNA structures of selected introns. For each chosen intron, an iterative procedure of searching for orthologous sequences and extending the predicted RNA structure resulted in a multiple sequence alignment, which was collapsed to a consensus sequence with a secondary structure. For each structure, the gene name and the length of the predicted structured region are shown. In the case of duplicated ribosomal gene introns where both paralogous introns share a similar structure, both gene names are given. Structure images were prepared using VARNA (Darty *et al.* 2009).

Since a predicted secondary structure of a single sequence is not generally sufficient to distinguish between a functional RNA and random sequences (Rivas and Eddy 2000), most RNA prediction methods require multiple homologous sequences. Thus our first step in RNA prediction was to identify orthologs of *S. cerevisiae* introns in other fungi. We searched for orthologs of intron-containing host genes in fully sequenced genomes and then for corresponding introns in those genes. In 36 fungal genomes, we were able to identify at least two orthologs for 281 introns and at least one ortholog for 305 introns (Figure 1A). Only the intron of YDR535C does not have an ortholog in any of the species searched, but annotation of this sequence as a gene is dubious. The vast majority of introns are conserved only in the *Saccharomyces* *sensu stricto*, and we identify fewer than four orthologs for each intron on average. In contrast, orthologs of yeast introns containing known intronic snoRNAs are found in a wider range of fungal genomes, having on average 9.6 orthologs (Figure 1A).

Orthologous intronic sequences were used to predict novel RNA structures using three independent computational methods: CMfinder, RNAz, and EvoFold. Each of these approaches yielded a very different number of predicted RNA structures in introns. RNAz, CMfinder, and EvoFold identified putative conserved RNA structures in 17, 54, and 175 introns, respectively. We found 14 structures in the intersection of all three approaches, within the only intron of each of *GLC7*, *HAC1*, *IMD4*, *MPT5*, *RPL18A*, *RPL18B*, *RPL22B*, *RPL28*, *RPS9A*, *RPS9B*, and *RPS13*, in the first intron of *RPL7A*, and in both introns of *RPS22B* (Figure 1B, File S1).

For further bioinformatic and experimental analysis, we decided to focus on the introns that had structures predicted by all three programs, together with their paralogs (*i.e.*, introns in *RPL22A* and *RPL7B*), and three introns with high prediction scores in at least two of the three approaches (*i.e.*, introns in *NOG2*, *YRA1*, and *PSP2*). We used the INFERNAL software (Nawrocki *et al.* 2009) and the Rfam library of covariance models to search the intron sequences for known ncRNA classes (Gardner *et al.* 2010). Besides the known snoRNAs, our other high-scoring RNA predictions do not resemble any previously known RNA families. We also used an iterative procedure combining covariance model searches using the INFERNAL package and manual inspection of multiple sequence alignments to identify additional homologs of our predicted structures in more distant species (Table 1, Figure 1C). We find that the known snoRNAs are very well conserved among Fungi: snR191 orthologs were found in all Saccharomycotina, snR44 in Saccharomycotina and Pezizomycotina, while snR54 is present in some Metazoan genomes as well as Fungi. The previously known *HAC1* intron–exon structure is also conserved in Fungi and Metazoa (Hooks and Griffiths-Jones 2011). Iterative INFERNAL searches for homologs of the predicted structures in the introns of *RPL18*, *RPL22*, *RPL7*, and *RPS9* allowed us to extend the conservation to the *Saccharomyces* and *Candida* clades. The identification of highly similar short motifs in the alignments of *RPL7*, *RPL18*, *RPL22*, *RPL28*, and *RPS9* introns demonstrated that predicted structures within introns are well conserved in *Saccharomyces* *sensu stricto*. The high sequence conservation means that relatively few compensatory mutations support these conserved predicted structures. Only in the case of the intron of *RPS13* is there evidence for maintenance of the RNA structure through multiple compensatory mutations.

**Table 1:** Conservation of RNA structure predictions

No.	Locus	Gene	Conservation
1	YBR189W	*RPS9B*	Saccharomycetaceae and *Candida* sp.
	YPL081W	*RPS9A*	
2	YDR064W	*RPS13*	Saccharomycetaceae
3	YDR381W	*YRA1*	Saccharomycetaceae
4	YER133W	*GLC7*	*Saccharomyces* *sensu stricto*
5	YFL031W	*HAC1*	Fungi and Metazoa
6	YFL034C-A	*RPL22B*	Saccharomycetaceae
	YLR061W	*RPL22A*	
7	YGL076C	*RPL7A*	Saccharomycetaceae and *Candida* sp.
	YPL198W	*RPL7B*	
8	YGL103W	*RPL28*	*Saccharomyces* *sensu stricto*
9	YGL178W	*MPT5*	*Saccharomyces* *sensu stricto*
10	YLR367W	*RPS22B*	Saccharomycetaceae except *L. lactis*
	5′ UTR		
11	YLR367W	*RPS22B*	Saccharomycotina and Pezizomycotina
	snR44		
12	YML017W	*PSP2*	*Saccharomyces* *sensu stricto*
13	YML056C	*IMD4*	Saccharomycetales and Diptera
	snR54		
14	YNL301C	*RPL18B*	Saccharomycetaceae and *Candida* sp.
	YOL120C	*RPL18A*	
15	YNR053C	*NOG2*	Saccharomycetaceae and *Candida* sp.
	snR191		

### Experimental detection of the predicted intronic ncRNA

In order to determine whether the predicted intronic RNAs are expressed and maintained in the cell, we performed RT-PCR on total and low molecular weight cDNA from haploid BY4741 WT *S. cerevisiae* strain. We designed three sets of specific primers to amplify the fragment of the intron with the predicted structure or snoRNA, the entire intron, and part of the exons flanking the intron. As a positive control for the PCR, we used the genomic DNA to amplify products with the primer pairs described above in the snR44, snR191, and snR54 snoRNA genes. Negative controls for all PCR experiments were performed with no template added to the reactions.

**Figure 2:**
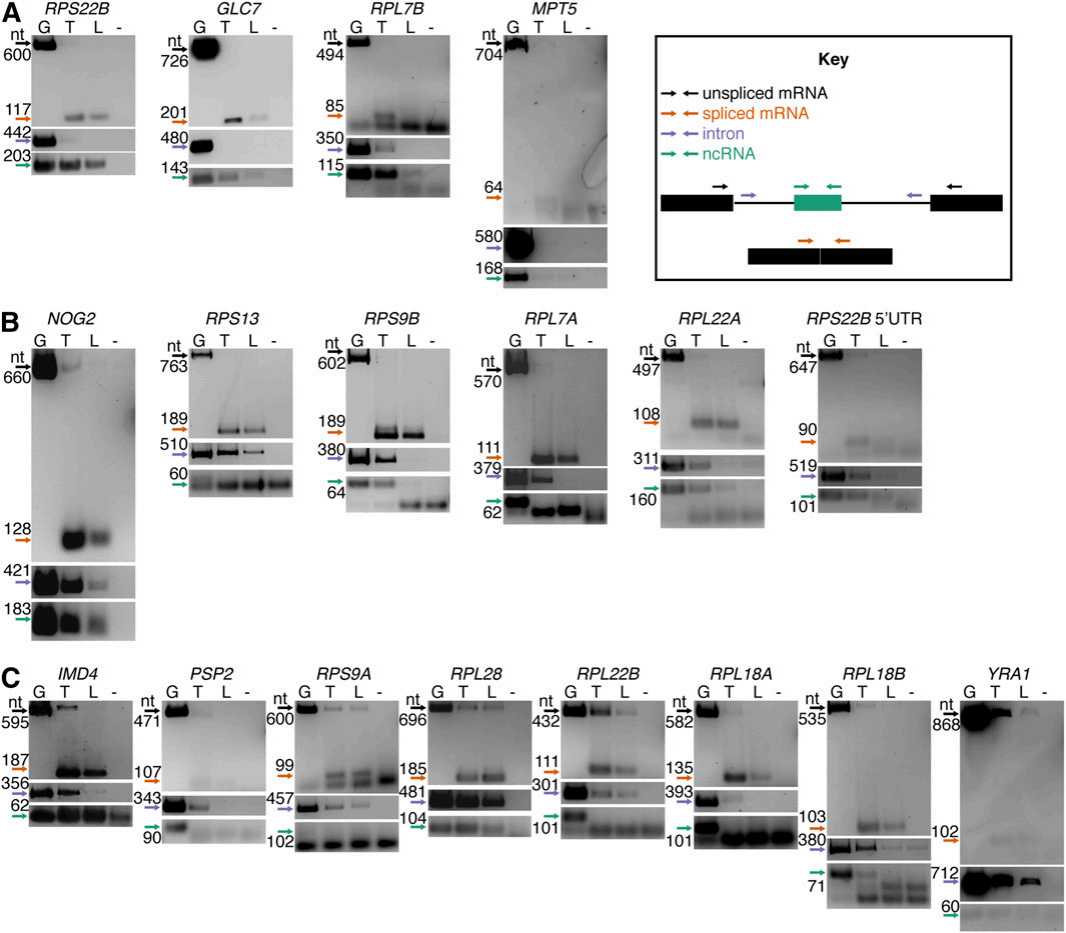
RT-PCR confirmation of intron fates conducted on total RNA and low-weight-enriched RNA using random priming. PCR of genomic DNA was used as positive control. (A) Agarose gel confirming ncRNAs expressed from introns and maintained in the cell. (B) Agarose gel confirming ncRNA expression accompanied by complete mRNA splicing. (C) Agarose gel confirming ncRNA expression accompanied by alternative splicing. Arrows indicate the expected size of the PCR products according to the key. Lane designations for DNA templates: G, Genomic DNA (positive control); T, total cDNA; L, low-molecular-weight-enriched cDNA; and −, no template negative control. With the exception of *GLC7* ncRNA, which was run on a separate gel, the images of mRNAs, introns, and ncRNAs for each gene were cropped from the same agarose gel picture with brightness and contrast applied equally across the entire image (full images available in Figure S1, Figure S2, Figure S3, Figure S4, Figure S5, and Figure S6). For small-size PCR products, cropping included primer dimers.

If a *bona fide* ncRNA product is processed from an intron, we expected to obtain bands corresponding to the spliced mRNA of the host gene, and also for the region of the predicted structure, but not the larger product from the complete intron. This pattern was observed for the *RPS22B* intron harboring the known snoRNA snR44 and for two other introns with predicted structures, namely, the intron of *GLC7* and the first intron of *RPL7B*, thus confirming these sequences as novel ncRNAs (Figure 2A, Figure S1). A similar pattern was also observed for *MPT5*, although its expression was very low (Figure 2A, Figure S1C). The RT-PCR corresponding to snR191 in *NOG2* displayed a pattern indicative of complete splicing of the host mRNA, but also showed the maintenance of the complete intron and the predicted ncRNA. We refer to these sequences as “introns maintained” in the cell after splicing as opposed to “introns retained” in the pre-mRNA. We observed a similar pattern of correct splicing with intact intron and ncRNA maintenance for our predicted structures in the introns of *RPS13*, *RPS9B*, *RPL7A*, *RPL22A*, and in the 5′ UTR intron of *RPS22B* (Figure 2B, Figure S2, Figure S3). These data show that a mixed population of mature ncRNAs and spliced but unprocessed introns are present in the cell. We also found that the mRNA transcripts of *IMD4* (containing snR54 in its intron), *PSP2*, *RPS9A*, *RPL28*, *RPL22B*, *RPL18A*, *RPL18B*, and *YRA1* were present in both spliced and unspliced forms, supporting the existing evidence that intron retention is the most common case of alternative splicing in yeast (Plass *et al.* 2012) (Figure 2C, Figure S4, Figure S5). We found no evidence of splicing of *HAC1* under the specific experimental conditions we tested (see Figure S6A). As negative controls, we conducted the same RT-PCR analysis on six intron-containing genes that had no predicted RNA structures. None of the six displayed patterns consistent with ncRNAs processed from the introns. The genes YBR219C and *BMH2* did not appear to be expressed and the four ribosomal protein genes *RPL27A*, *RPS27B*, *RPS16A*, and *RPS19B* showed the intron-maintained pattern (Figure S6, B–G). RNA-seq data show higher expression of introns in ribosomal protein genes that contain predicted RNA structures.

We also validated the presence of expressed intronic sequences in the cell by reanalyzing deep sequencing data of total RNA extracted from *S. cerevisiae* (Hooks *et al.* 2014). We counted all reads overlapping introns and normalized by the intron length and the total number of reads mapped to obtain RPKM values. Median intron expression was 15.3 RPKM. We observed that 17 of 19 introns with predicted structures have evidence of expression (more than 150 reads or 20 RPKM), seven of which fell within the 90th percentile of intron expression.

**Figure 3:**
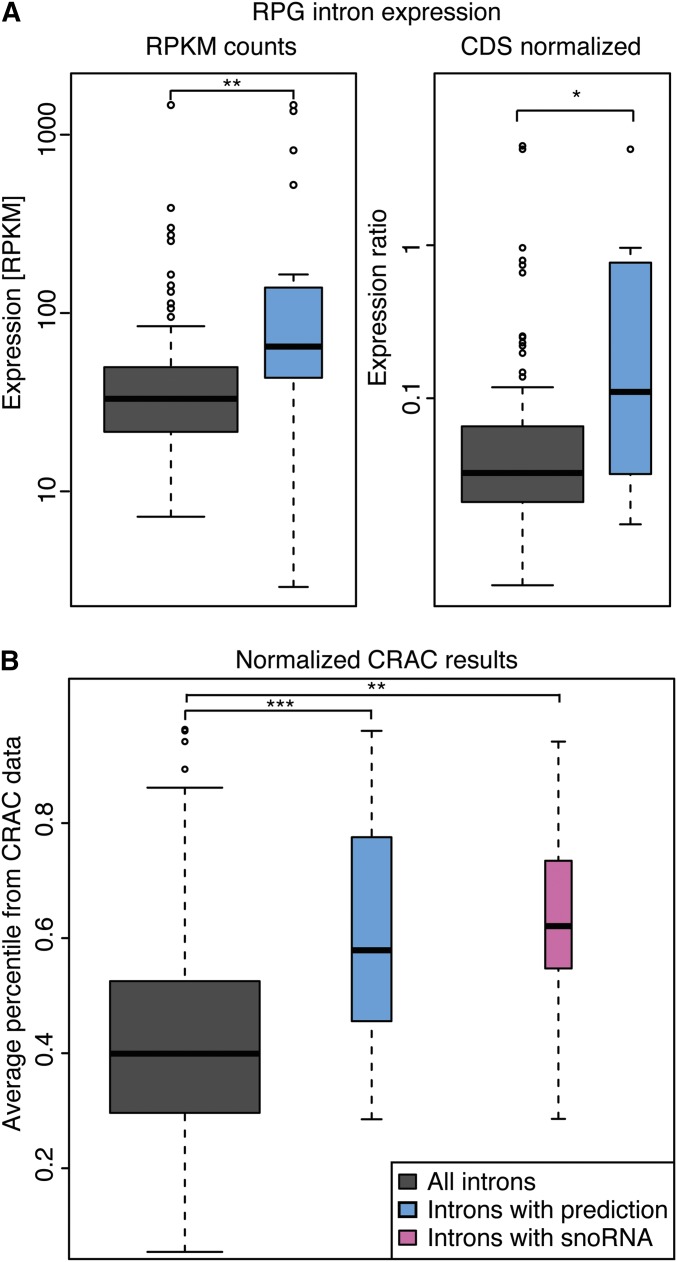
Properties of loci containing intronic novel RNA predictions or snoRNAs. Quantifications were based on RNA-seq data of the same total RNA as used in Figure 2 (GEO accession no. GSE58884) and on CRAC data (GEO accession no. GSE40046). (A) Box plots showing ribosomal protein intron transcript levels in RPKM without normalization and with normalization to host gene transcript levels. Values are shown for all RP gene introns and 12 RP gene introns with predictions. (B) Association of introns with the exosome. We reanalyzed 16 independent sequencing experiments by Schneider *et al.* (2012) of RNA fragments cross-linked to exosome components. Reads mapping to each intron were normalized by the host gene expression estimated by RNA-seq. For each CRAC experiment an intron was given a percentile value of how frequently it was bound to an exosome component compared to other introns. Values presented are averaged percentile derived from 16 experiments and are shown for all introns, the 19 introns with predictions, and the eight introns containing snoRNAs. The levels of significance for the Mann–Whitney *U*-tests are represented as follows: *** *P* < 10^−3^; ** *P* < 0.01; * *P* < 0.05.

Since a third of all introns are found in highly expressed ribosomal protein (RP) genes, we next measured the relative expression levels of maintained introns in RP that contain or do not contain predicted structures. Since the mRNA levels of the genes hosting predicted intronic structures appeared to be greater than the average mRNA amount for the entire set of intron-containing genes, we also normalized the intron expression levels by the level of their host gene transcript. Interestingly, expression of RP introns with predictions was significantly higher compared with all RP introns before and after normalizing for host gene expression (median RPKM 65.5 compared to 33.0; Mann–Whitney *U*-test *P*-value = 0.006 and median normalized expression 0.11 compared to 0.03; Mann–Whitney *U*-test *P*-value = 0.014) (Figure 3A).

### Introns with predicted RNA structures are more likely to be targeted by the exosome

We hypothesize that introns with RNA structures either contain novel ncRNAs or are involved in the regulation of pre-mRNA splicing; in both cases, the host transcript would be expected to associate with the exosome complex. We analyzed the data of Schneider *et al.* (2012), who used *in vivo* RNA cross-linking (CRAC) of exosome components to show that noncoding RNAs, snoRNAs, pre-tRNAs, and pre-mRNAs are the most prominent exosome targets. The Schneider *et al.* (2012) data set (GEO accession no. GSE40046) contains deep sequencing reads from 16 separate *in vivo* cross-linking experiments to the tagged protein components of the exosome. When we normalized the number of reads of each intron-containing gene from GSE40046 by the average number of reads for this intron-containing gene from GSE58884, we observed that the set of genes with both known snoRNA host genes and our novel RNA predictions appears to be targeted by the exosome machinery more than expected (our predictions, Mann–Whitney *U*-test, *P* = 2.44 × 10^−4^; snoRNAs, *P* = 0.005) (Figure 3B). This suggests that the introns of interest are either retained in pre-mRNAs or contain noncoding RNAs. Taken in conjunction with our RT-PCR data, the CRAC data indicated that the pre-mRNA transcripts of *PSP2*, *RPS9A*, *RPL18A*, and *RPL18B* are maintained in the cell, possibly due to the stable secondary structures that are resistant to the degradation by exosome. In contrast, the presence of a novel ncRNA was indicated for the *MTP5* intron.

### Function of the *GCL7* intronic ncRNA

**Figure 4:**
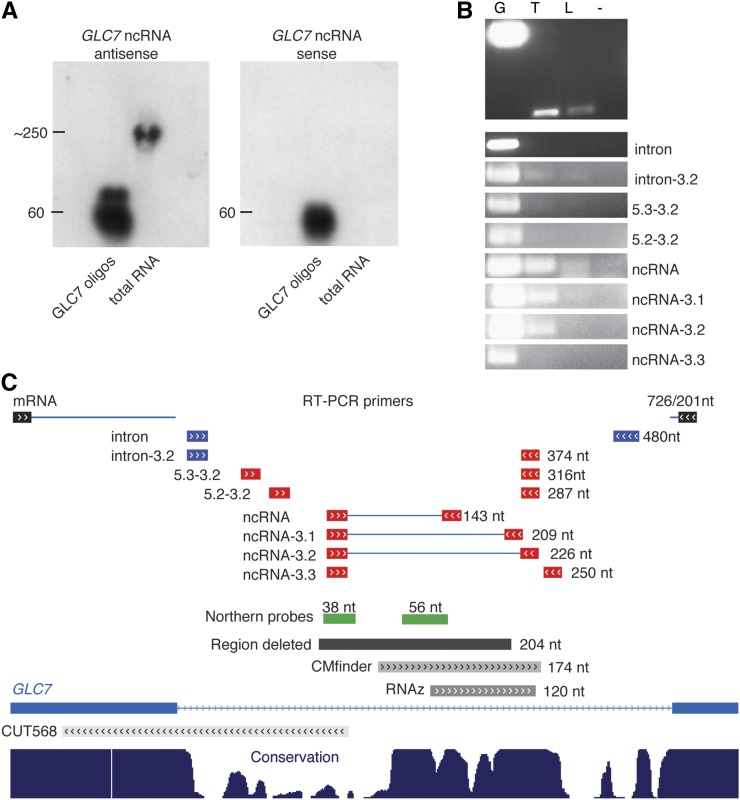
Characterization of the size and expression of the ncRNA within *GLC7* intron. (A) Northern blot of *S. cerevisiae* total RNA and oligos mimicking *GLC7* intron. The blots were probed with strand-specific probes showing expression of ncRNA in antisense (left panel) and sense orientation (right panel) to the *GLC7* gene. The estimation of the sizes is based on the 60-nt oligos visible on the film and a comparison with the markers visible on the membrane. (B) RT-PCR on total RNA and low-weight-enriched RNA using random priming showing the expression of ncRNA within the *GLC7* intron. PCR of genomic DNA was used as positive control. Names of the primers are listed on the right and are the same as indicated in C. Gel annotation: G, genomic DNA; T, total cDNA; L, low-molecular-weight-enriched cDNA; and −, no template negative control. (C) Data uploaded into the University of California Santa Cruz genome browser for sequence annotation and data visualization presenting annotated *GLC7* intron. Primer names used for RT-PCR are listed next to black, blue, and red boxes indicating their position with respect to the gene annotation below and the size of the corresponding PCR product. Lines joining primers mark the amplified sequences; the regions targeted for deletion by the loxP method are symbolized by the black box; the location of the strand-specific Northern probes are shown in green; and regions with the putative structure predicted by RNAz and CMfinder are shown in gray. All features map directly onto the fragment of the gene structure diagrams. The bottom panel represents the degree of conservation of gene regions among seven yeast species.

The intronic sequence in *GLC7* was recently shown to play a role in the cellular response to osmotic pressure (Parenteau *et al.* 2008). We therefore investigated further the putative ncRNA derived from this intron. First, we used Northern hybridization to confirm the RT-PCR analysis of expression of the ncRNA and confirm the strand from which it is expressed, since the computational predictions indicated that the stable RNA structure can be formed by this *GLC7* intron region in both orientations. Strand-specific probes suggest expression of a 250-nt molecule from the antisense strand of the *GLC7* intron (Figure 4A, left panel). Second, we employed primer walking to define the 5′ and 3′ ends of the ncRNA (Figure 4B). We were able to specifically define *GLC7* ncRNA boundaries, which are different from those of the previously annotated CUT568, which is also antisense to the *GLC7* gene (Figure 4C). The sizes and positions of PCR products, Northern probes used, and computational predictions are shown in Figure 4C.

**Figure 5:**
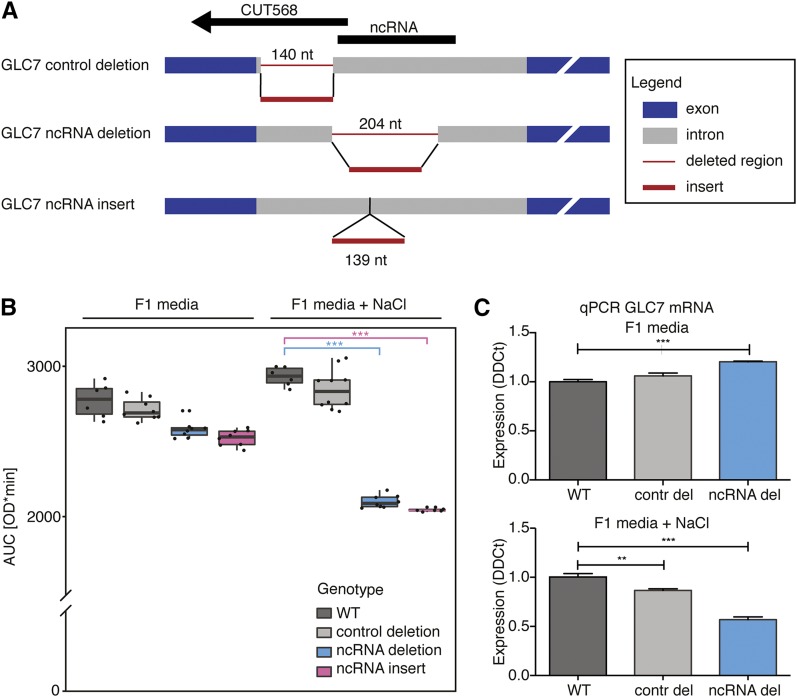
Effects of GLC7 intron mutation. (A) Schematic representation of intron mutants used for phenotype studies. (B) The GLC7 ncRNA deletion and GLC7 ncRNA insertion mutants disrupting the structure sequence of the GLC7 intron were compared with the WT strain and the intronic negative control in F1 medium containing 0.9 M NaCl. Values in the box plots present the means of the AUC as determined by the R pracma package. Significance estimated by one-way ANOVA with Dunnett’s multiple comparison test (*** *P* < 0.0001). (C) Expression levels (average expression with SEM) of GLC7 mRNA in the WT, control mutant, and the GLC7 ncRNA deletion mutant grown in F1 and F1 + 0.9 M NaCl media, assessed by RT-qPCR. Significance estimated by one-way ANOVA with Dunnett’s multiple comparison test (*** *P* < 0.0001, ** *P* = 0.005).

To study the phenotype of the ncRNA, we used two approaches: deletion and alteration of the ncRNA structure, the latter of which increases the distance between the two predicted hairpins. We deleted two regions in the intron of *GLC7* via PCR-mediated gene deletion and the cre-loxP system: an intronic region overlapping with CUT568 but not with the ncRNA (negative control deletion mutant) and the intronic region corresponding to the ncRNA (*GLC7* ncRNA deletion mutant). For the alteration, we inserted 139 bp in the middle of the predicted ncRNA to modify its structure (*GLC7* ncRNA insertion mutant; see *Materials and Methods*). Previously it was shown that replacing the *GLC7* gene with its cDNA decreases the cell viability in NaCl stress (Juneau *et al.* 2006; Parenteau *et al.* 2008). In order to determine whether this defect was due to the ncRNA structure rather than the intron in itself or the CUT568, we tested all engineered strains with the mutated *GLC7* intron in F1 medium and in F1 medium containing 0.9 M NaCl (Figure 5A). In F1 supplemented with 0.9 M NaCl, we observed a significant difference in growth, as estimated by the AUC (Norris *et al.* 2013), for both *GLC7* ncRNA deletion and insertion mutants compared with the WT (one-way ANOVA with Dunnett’s multiple comparison test: for both mutants *P* < 0.0001) or the negative control deletion (for both mutants *P* < 0.0001; Figure 5B). Specifically, we found that in the F1 + 0.9 M NaCl media, the lag phase (λ) is longer in the *GLC7* ncRNA deletion and insertion mutants. Moreover, their maximum growth rate (μ) and the final biomass after 48 hr (A) are decreased, whereas for the negative control deletion mutant those parameters are the same as the WT (Figure S7). We attempted to restore the fitness of the *GLC7* ncRNA deletion mutant by inserting the ncRNA expressed under the strong constitutive promoter (see *Materials and Methods*). There was no significant recovery in fitness from the addition of the sense or antisense orientation ncRNA overexpression plasmids (Figure S8). This suggests that the intronic ncRNA is exclusively *cis*-acting.

We further verified the phenotypic difference of the mutant using one-to-one competition experiments. The WT BY4742 strain and the *GLC7* deletion mutant were grown together in F1 medium supplemented with 0.9 M NaCl, and the composition of the population was analyzed after 37 generations. A significant drop in relative amount of the mutant strains was detected with just over 25% of total cells being *GLC7* deletion mutant, instead of the expected 50% (Student’s *t*-test, *P* = 7.30 × 10^−5^). Taken together, these data further indicate that the deletion of the putative ncRNA in the *GLC7* intron is responsible for the observed impairment of cell growth rate and competitive fitness during salt stress, whereas deletion of the upstream intronic region overlapping the CUT568 expressed from the opposite strand has no impact on the phenotype in salt stress.

We then looked at the expression of *GLC7* via real-time PCR in WT, control deletion mutant, and *GLC7* ncRNA deletion mutant. The data show that the deletion of the ncRNA or the control region in *GLC7* intron does not impair *GLC7* expression in F1 media. In fact, the *GLC7* mRNA level in ncRNA deletion mutant appears to be slightly elevated compared to that of WT or control deletion (mean ± SEM: 1.20 ± 0.01). However, in salt stress the *GLC7* mRNA level of the *GLC7* ncRNA deletion mutant is reduced to 0.57 ± 0.03 of that of the WT (Figure 5C), whereas the *GLC7* mRNA level in control deletion decreases only by a small amount (mean ± SEM: 0.87 ± 0.02). We suggest that the phenotypic effect of the ncRNA knockout under salt stress may be due to the decreased expression or a splicing defect of *GLC7* in the *GLC7* ncRNA deletion mutant.

## Discussion

ncRNAs can be present in intergenic regions (David *et al.* 2006), inside exons, as in the case of ncRNA derived from *TRM10* mRNA (Pircher *et al.* 2014), or within introns (Ooi *et al.* 1998; Nakaya *et al.* 2007). Using multiple computational methods, we predicted stable and conserved RNA structures in 19 introns, 12 of which are present in RP genes. By RT-PCR, we validated the presence of the predicted ncRNAs and, in several cases, of the whole introns. Our predicted RNA structures include in the 5′ UTR intron of *RPS22B* (Figure 2B) and in the intron of *RPL18A* (Figure 2C). Both introns have been previously reported to trigger RNase III-mediated mRNA degradation by Rnt1p (Danin-Kreiselman *et al.* 2003). We also predicted and validated the expression of putative ncRNAs in the introns of *RPS9A* and *RPS9B* (Figure 2, B and C), and these introns were previously shown to regulate expression of both their host genes and their paralogs (Plocik and Guthrie 2012). In another study on the effects of intron deletion (Parenteau *et al.* 2011), 11 RP introns besides *RPS13* were shown to regulate the expression of the host gene or its paralogous copy, change cell sensitivity to drugs, or alter the competitive fitness when deleted. Our RNA-seq data analysis shows that introns in RP genes containing RNA structures are significantly more expressed than the introns in RP genes lacking a predicted ncRNA. Among the seven non-RP proteins with high-scoring predictions, only the *PSP2* introns have no previously suggested function. *HAC1* and *YRA1* introns contain structures that regulate their own splicing and *IMD4* and *NOG2* contain snoRNAs that guide chemical modification of other RNAs, whereas *GLC7* and *MPT5* introns appear to be required for stress tolerance (Parenteau *et al.* 2008). Our data raise the possibility that, at least in some cases, an intronic RNA structure is responsible for the biological function, rather than the intron itself.

Our experimental characterization and follow-up analysis have allowed us to generate hypotheses regarding the mechanism of function of specific sequences. For example, the *GLC7* intron was previously shown to mediate the response to salt stress (Parenteau *et al.* 2008) and we now demonstrate that the factor responsible for the biological function is an intronic sequence with a discreet RNA structure (Figure 5). Furthermore, characterization of the stable ncRNA by RT-PCR and Northern combined with unsuccessful rescue experiment suggests that the predicted structure functions in *cis*. RNAs functioning in *cis* are common among eukaryotes and originate from their own transcriptional unit (Quinn and Chang 2016). There is a prevalence of antisense ncRNA transcripts across the yeast (Neil *et al.* 2009) and mammalian genomes (Core *et al.* 2008) and such ncRNAs are able to regulate the expression of the gene on the opposite strand in a variety of ways including: transcriptional interference (Houseley *et al.* 2008; Hainer *et al.* 2011), alternative splicing (Wang *et al.* 2005), and at the translational level (Carrieri *et al.* 2012).

New techniques, such as *in vivo* RNA cross-linking to protein complexes coupled with next-generation sequencing, can aid the discovery of novel pathways involving all types of ncRNA, including those encoded in introns. Our analysis of exosome target data presented by Schneider *et al.* (2012) indicates genes with intronic RNA structure predictions are more likely to be transcriptionally regulated or contain novel ncRNAs.

With the exception of a few snoRNAs, *S. cerevisiae* introns do not appear to contain classical intronic ncRNAs. Although the function of most intronic RNAs in higher eukaryotes is still unknown, the evidence of tissue-specific expression (Louro *et al.* 2008) and binding to protein complexes known to promote epigenetic modifications (Guil *et al.* 2012) indicates that intronic transcripts may have specific functions, rather than arising from spurious transcription or slow pre-mRNA turn-over. Our results provide clear evidence for the function of the *GLC7* intronic RNA structure in an intron-poor single-celled fungal species.

Our work, and that of others mentioned here, raises interesting questions about the general nature of intron stability postsplicing. Introns removed by splicing have been thought to be rapidly degraded and the main focus of intron biology has concerned elucidation of splice sites and the arrangement of the splicing machinery. For example, it was observed that the deletion of the debranching enzyme promotes the accumulation of lariat introns (Chapman and Boeke 1991), so it was assumed that all introns are rapidly debranched and targeted for degradation in normal cells. Tiling arrays and next generation sequencing have readily shown that some intronic sequences are abundant in the cell, but they are usually dismissed as remnants of normal splicing or part of immature pre-mRNAs (Louro *et al.* 2009). Recently, the fate of introns themselves has been systematically assessed in *Xenopus tropicalis* embryos showing that 90% of introns are maintained in the nucleus postsplicing (Gardner *et al.* 2012) and ∼5% of genes generate lariats that are stable in the cytoplasm (Talhouarne and Gall 2014). Linear intron-derived ncRNAs with some similarities to snoRNAs have been found in HeLa cells and human embryonic stem cells (Yin *et al.* 2012). Most importantly, circular intronic RNAs increase the expression of their host genes as exemplified by gene *ANKRD52* (Zhang *et al.* 2013). We and others observe that many of the introns examined are retained in the pre-mRNA or maintained in the cell after splicing. This is consistent with the observations of Coleclough and Wood (1984) who were the first to described discrete intron products processed from the mouse immunoglobin pre-mRNA. Immunoglobulin mRNAs are expressed at high levels, like the ribosomal genes tested in our study. We therefore speculate that postsplicing intron stability might be a prevalent phenomenon for highly expressed genes and that intron products may be regulators of these highly expressed gene products.

### Conclusions

We undertook a systematic approach using the well-studied *S. cerevisiae* genome in order to identify introns with undiscovered function. Comparing intron sequences of related yeast species, we found at least 19 introns contain putative conserved RNA structures. By RNA-seq and RT-PCR, we show that several of the intronic sequences containing secondary structures are not degraded after removal from pre-mRNAs. Furthermore, we show that RNA structures embedded in introns can be directly responsible for regulating gene expression and maintaining phenotype in the intron-poor yeast. For example, a small portion of the *GLC7* intronic sequence, representing a novel RNA structure, plays an important role in the cellular response to salt stress. More generally, the cellular abundance of intron sequences from RP genes with predicted intronic RNAs is significantly higher than for those lacking such predictions. Overall, our data support the possibility that the presence of functional RNA structures in introns has contributed to selective intron retention in the *Saccharomycetes*.
